# Using stepwise regression to address multicollinearity is not appropriate

**DOI:** 10.1097/JS9.0000000000001208

**Published:** 2024-02-16

**Authors:** Wen-Feng Xi, Qing-Wei Jiang, Ai-Ming Yang

**Affiliations:** Department of Gastroenterology, State Key Laboratory of Complex Severe and Rare Diseases, Peking Union Medical College Hospital, Peking Union Medical College and Chinese Academy of Medical Sciences, Beijing, People’s Republic of China

*Dear Editor*,

The recent online publication of the study by Ning *et al*.^1^ in the *International Journal of Surgery* has captured our attention, and we have read this with great interest. This prospective cohort study focused on predicting survival in patients with infected pancreatic necrosis (IPN). The authors utilized the Random Survival Forest (RSF) method to unveil seven pivotal predictors related to mortality, highly aligned with clinical experience. Besides, the construction of a Conditional Survival Nomogram promises real-time survival insights, offering great support for clinical decision-making processes. We deeply appreciate the significance of this study; however, we have some suggestions for consideration, mainly regarding statistical strategies for addressing multicollinearity.

First, correlation analysis is a common method for assessing multicollinearity, and its applicable conditions need to be strictly observed. Before conducting Cox regression multivariate analysis, the authors included seven variables in Spearman rank correlation analysis to assess multicollinearity. This type of correlation analysis is suitable for skewed distribution data and ordinal categorical variables. However, the authors mistakenly included binary variables, such as hemorrhage, in the correlation analysis, resulting in a correlation coefficient that lacked meaningful interpretation.

Second, we express reservations about the claim that stepwise regression can handle multicollinearity. When the authors identified multicollinearity among variables with correlation analysis, the paper continued with, ‘*thus, we performed a stepwise backward Cox regression analysis to further control for collinearity.*’ This might not be appropriate. The essence of stepwise regression is to simplify the model and avoid overfitting, rather than addressing multicollinearity. Multicollinearity should be mitigated prior to conducting stepwise regression; otherwise, the effects of individual variables may be challenging to accurately estimate. Multicollinearity leads to unstable estimation of model parameters, increasing the standard errors of parameter estimates. Stepwise regression may consequently remove variables that are actually important but whose statistical significance is affected by multicollinearity.

Third, methods to address multicollinearity. The Variance Inflation Factor (VIF) may be calculated to assess multicollinearity^2^ (if VIF >5 or 10) and then remove relevant variables. Additionally, in Cox regression, regularization methods such as ridge^3^ or Lasso regression^4^ can be employed. These techniques reduce the impact of multicollinearity by adding penalty terms to the regression coefficients. While Cox models with regularization are more complex than those without, the computational process remains transparent. We can understand how the model is constructed and how the regularization terms influence coefficient estimation, allowing for interpretation within clinical contexts. In this study, there were 25 candidate variables, with only 80 cases of the endpoint event (death). The authors employed a strategy of ‘RSF - Cox univariate - Cox multivariate’ to reduce dimensionality and then construct a nomogram model. If a Cox model with regularization is used, the first two steps can be consolidated into one, enhancing modeling efficiency and mitigating the issue of multicollinearity.

To conclude, this is an undoubtedly meaningful and sincere study that unveils the mortality prediction factors and models survival status for patients with IPN. Here, we presented our humble suggestions on the handling of multicollinearity. It is not uncommon to mistakenly place hope on stepwise regression to address multicollinearity. We anticipate that there will be improvements in the stability and accuracy of the predictive model after resolving multicollinearity.

## Ethical approval

Not applicable.

## Consent

Not applicable.

## Sources of funding

This work was supported by the National Key Clinical Specialist Construction Project (Grant No. ZK108000) and the National Key R&D Program of China (Grant No. 2022YFC3602103).

## Author contribution

W.-F.X.: conceptualization and writing – original draft; Q.-W.J. and A.-M.Y.: writing – review and editing.

## Conflicts of interest disclosure

All authors declare that there are no conflicts of interest.

## Research registration unique identifying number (UIN)

Not applicable.

## Guarantor

The guarantor is the corresponding author, Prof. Ai-Ming Yang.

## Provenance and peer review

Not applicable.

## Data availability statement

Not applicable.

## Provenance and peer review

Not applicable.
